# The Construction of Sodium Alginate/Carboxymethyl Chitosan Microcapsules as the Physical Barrier to Reduce Corn Starch Digestion

**DOI:** 10.3390/foods13091355

**Published:** 2024-04-28

**Authors:** Linjie Zhan, Zhiwei Lin, Weixian Li, Yang Qin, Qingjie Sun, Na Ji, Fengwei Xie

**Affiliations:** 1 College of Food Science and Engineering, Qingdao Agricultural University, Qingdao 266109, China; zlj980606@163.com (L.Z.); lzwhappy2021@163.com (Z.L.); 18234566016@163.com (W.L.); qinyjnu@163.com (Y.Q.); phdsun@163.com (Q.S.); 2 Qingdao Special Food Research Institute, Qingdao 266109, China; 3 Department of Chemical Engineering, University of Bath, Bath BA2 7AY, UK; fwhsieh@gmail.com

**Keywords:** carboxymethyl chitosan, sodium alginate, citric acid, resistant starch

## Abstract

To enhance the resistant starch (RS) content of corn starch, in this work, carboxymethyl chitosan/corn starch/sodium alginate microcapsules (CMCS/CS/SA) with varying concentrations of SA in a citric acid (CA) solution were designed. As the SA concentration increased from 0.5% to 2%, the swelling of the CMCS/CS/SA microcapsule decreased from 15.28 ± 0.21 g/g to 3.76 ± 0.66 g/g at 95 °C. Comparatively, the onset, peak, and conclusion temperatures (*T*_o_, *T*_p_, and *T*_c_) of CMCS/CS/SA microcapsules were higher than those of unencapsulated CS, indicating that the dense network structure of microcapsules reduced the contact area between starch granules and water, thereby improving thermal stability. With increasing SA concentration, the intact and dense network of CMCS/CS/SA microcapsules remained less damaged after 120 min of digestion, suggesting that the microcapsules with a high SA concentration provided better protection to starch, thereby reducing amylase digestibility. Moreover, as the SA concentration increased from 0.5% to 2%, the RS content of the microcapsules during in vitro digestion rose from 42.37 ± 0.07% to 57.65 ± 0.45%, attributed to the blocking effect of the microcapsule shell on amylase activity. This study offers innovative insights and strategies to develop functional starch with glycemic control properties, holding significant scientific and practical value in preventing diseases associated with abnormal glucose metabolism.

## 1. Introduction

Changes in dietary habits and lifestyles have contributed to the prevalence of type 2 diabetes, making it a significant threat to human health and life safety [1]. Type 2 diabetes arises when the body fails to properly absorb excessive glucose, leading to elevated blood sugar levels. Consequently, individuals with type 2 diabetes must meticulously manage their blood sugar levels and adhere to more stringent dietary restrictions compared to those without the condition.

Starch, one of the three essential nutrients, serves as the primary source of energy for humans and is integral to daily life [2]. However, the rapid release of glucose following starch digestion in the gastrointestinal tract can cause a sharp increase in blood sugar levels, contributing to chronic metabolic disorders such as type 2 diabetes, cardiovascular diseases, and hyperglycemia [3]. Thus, mitigating the rate and extent of starch digestion in starchy foods could help prevent sudden spikes in blood glucose levels, thereby promoting postprandial blood glucose stability.

In recent years, researchers have proposed a variety of strategies to regulate the digestion rate of starch in response to the dietary needs of individuals with type 2 diabetes who consume starch-based food. These methods primarily include chemical, enzymatic, and physical embedding approaches [4,5]. Enzymatic modification typically involves catalytic hydrolysis using enzymes like pullulanase or modifying the molecular structure of starch with transglycosidase. However, the stringent requirements for enzyme temperature and pH can lead to unstable product yields, and the high cost of enzymes hinders industrial production [6,7,8]. Chemical methods involve replacing hydroxyl groups in starch chains through processes such as oxidation, esterification, and etherification. Despite their potential, chemical methods suffer from drawbacks such as prolonged production times, variable product quality, low reaction rates, and the risk of environmental pollution [9,10,11,12]. Physical embedding, on the other hand, entails directly shielding starch particles to prevent contact with digestive enzymes [13]. This method offers advantages such as environmental friendliness, easy access to raw materials, and the simplicity of operation. Consequently, physical encapsulation holds significant promise for reducing the rate and extent of starch digestion. Recent research efforts have focused on exploring the encapsulation characteristics and protective mechanisms of various wall materials, including sodium alginate, guar gum, pectin, chitosan, konjac gum, and other non-starch polysaccharides [14,15,16]. For instance, Ning et al. developed a konjac glucomannan (KGM)-coated dendrimer corn starch (DCS) composite material, demonstrating that KGM effectively hindered the digestion of DCS by digestive enzymes [17,18,19]. Similarly, Feng et al. [20] investigated the inclusion of monimus β-glucan (HEBG) in wheat starch, revealing that HEBG effectively inhibited the digestion rate of starch due to its protective effect of HEBG on wheat starch.

Carboxymethyl chitosan (CMCS) is an amphoteric polyelectrolyte containing cationic -NH^3+^ and anionic -COO^−^ groups, offering favorable biocompatibility and water solubility. Similarly, sodium alginate (SA) is rich in -COO^−^ and hydroxyl groups, and its aqueous solution exhibits adhesive properties. Consequently, SA and CMCS find extensive use in biological materials owing to their excellent characteristics, including biocompatibility, easy degradation, and abundant sources [21,22,23]. However, pure SA and CMCS hydrogels lack good mechanical properties and functionality, which limits their applications [18,24]. Citric acid (CA) emerges as a promising crosslinker due to its low cytotoxicity, cost-effectiveness, and widespread natural occurrence. Structurally, CA is a tricarboxylic acid compound with three -H^+^ groups, facilitating the formation of gel networks by strengthening hydrogen bonds [25,26,27,28,29].

Utilizing the intrinsic properties of CMCS, SA, and corn starch (CS), we employed these materials as raw ingredients to encapsulate CS via physical crosslinking, aiming to mitigate starch digestibility. Comprehensive analyses, including microstructure examination, solubility testing, swelling assessments, scanning electron microscopy (SEM), differential scanning calorimetry (DSC), and in vitro digestion studies of the microcapsules, were conducted to elucidate their structure, gelatinization, and digestion characteristics. The findings reveal that the microcapsules enhanced the thermal stability and heat resistance of CS while reducing its digestibility. Consequently, this study is of great significance for stabilizing blood glucose levels and alleviating and preventing blood glucose metabolism problems in humans.

## 2. Materials and Methods

### 2.1. Materials

Corn starch (CS) with a molecular weight of 3.43 × 10^8^ Da and an amylose content of 23.67% (Zhucheng Xingmao Corn Developing Co., Ltd., Weifang, China). Carboxymethyl chitosan (Shanghai Macklin Biochemical Technology Co., Ltd., Shanghai, China). Sodium alginate of medium viscosity (Qingdao Bright Moon Seaweed Group Co., Ltd., Qingdao, China). Citric acid (Sinopharm Chemical Reagent Co., Ltd., Shanghai, China). Enzymes α-glucosidase and pancreatin, derived from porcine pancreas (Sigma-Aldrich Chemical Co., Ltd., St. Louis, MO, USA). Glucose oxidase–peroxidase (GOPOD) assay kits (K-GLUC) (Megazyme International Ireland Co., Ltd., Wicklow, Ireland). All other chemicals were of analytical quality and were utilized as received.

### 2.2. Preparation of CMCS/CS/SA Microcapsules

The CMCS/CS/SA microcapsules were fabricated following the physical crosslinking method outlined by Jing et al. [30]. In brief, SA (0.5 g, 1 g, 1.5 g, and 2.0 g) was dissolved in 100 mL of distilled water to obtain 0.5%, 1%, 1.5%, and 2% concentrations, respectively. Concurrently, CMCS (1 g) was dissolved in 100 mL of distilled water to obtain a 1% CMCS solution. The SA solution and the CMCS solution were thoroughly mixed with ungelatinized CS (15%) for 1 h at 25 °C to form a homogenous mixture. Subsequently, the resulting mixture of CMCS, SA, and CS was gradually added dropwise into a CA solution (0.5% *w*/*w*, 50 mL, pH~2.5) using a syringe while stirring continuously for 3 h to ensure the complete reaction and crosslinking of SA and CMCS under CA conditions for starch encapsulation. The resulting CMCS/CS/SA microcapsules were washed three times with distilled water and then dried at 45 °C to obtain the dried samples. The microcapsules prepared using different SA concentrations (0.5%, 1%, 1.5%, and 2%) are denoted as CMCS/CS/SA_0.5%_, CMCS/CS/SA_1%_, CMCS/CS/SA_1.5%_, and CMCS/CS/SA_2%_, respectively.

### 2.3. Texture Profile Analysis of CMCS/CS/SA Microcapsules

The flat-bottom cylindrical probe P36R was utilized to evaluate both fresh microcapsules that had just been cured in CA solution and CMCS/CS/SA microcapsules in a boiling water bath for 30 min. During testing, the probe descended at a rate of 1.0 mm/s, ascended at 1.0 mm/s, and applied a deformation force of 50%. To enhance detection accuracy, 10 microcapsules were randomly selected for a single assessment, and the average value was computed based on three repeated tests.

### 2.4. Characterization of CMCS/CS/SA Microcapsules

The analysis of CMCS/CS/SA microcapsules was carried out using modified procedures from reference [31]. Observations of the outer surface and cross-sectional views of the dried microcapsules and microcapsules after digestion in vitro were made using a scanning electron microscope (SEM) (Model S-3400 N, Hitachi Instrument Co., Ltd., Tokyo, Japan). For the SEM examination, the samples received a triple coating of gold.

### 2.5. Measurement of Swelling Power (SP) and Solubility (S) of CMCS/CS/SA Microcapsules

The method for determining the swelling power (SP) and solubility (S) of CMCS/CS/SA microcapsules was adapted from the literature with minor adjustments [32]. Specifically, about 200 mg of CMCS/CS/SA microcapsules (on a dry basis) were submerged in about 20 mL of water and boiled at different temperatures of 55 °C, 65 °C, 75 °C, 85 °C, and 95 °C for 30 min. After boiling, the microcapsules were promptly cooled to room temperature in an ice bath and then centrifuged at 3000 rpm (AnkeLXJ-IIB centrifuge, Shanghai, China) for 15 min. Following centrifugation, the supernatant was cautiously decanted and preserved, while the residue was weighed to determine the SP. The supernatant was transferred from the tube into a pre-weighed glass dish. The glass dish containing the supernatant was then dried to a constant weight at 105 °C and weighed again. The percentages of the SP and S (%SOL) were calculated using the following equation:

%SOL = A/S × 100(1)

SP = (B × 100)/S(100 − %SOL)(2)

In the formula, %SOL represents the percentage solubility of the test sample, SP denotes the swelling power of the test sample, A is the mass of the soluble substances remaining after the test sample has been completely dried, B is the mass of the solid precipitate remaining at the end of the experiment, and S is the mass of the microcapsules after drying.

### 2.6. Thermal Properties of CMCS/CS/SA Microcapsules

The thermal properties of fresh CMCS/CS/SA microcapsules were analyzed using a differential scanning calorimeter (Mettler Toledo, Schwerzenbach, Switzerland) [16]. Microcapsule specimens, weighing from 3.0 to 6.0 mg, were placed in an airtight aluminum pan (Mettler Toledo, Schwerzenbach, Switzerland, 40 μL, 99.5% Al) and mixed with an excess of water at a 1:2 ratio. The CMCS/CS/SA microcapsules were equilibrated for 12 h before their temperature was increased from 25 °C to 125 °C in a nitrogen environment at a rate of 10 °C/min, then cooled back to 25 °C upon heating completion. The onset temperature (*T*_o_), peak temperature (*T*_p_), end temperature (*T*_c_), and the enthalpy change (*ΔH*) were documented throughout. Starch content (on a dry basis) was used for calculating the sample weight.

### 2.7. Measurement of Total Starch Content

The procedure was conducted with minor adjustments [33]. Initially, approximately 2 g of the microcapsules was measured, pulverized, and sieved through a 40-mesh screen. The obtained powder underwent three ethyl ether rinses (30 mL each) to extract fats, followed by triple rinsing with 150 mL of 85% ethanol to remove soluble sugars. Subsequently, the residue was dissolved in 100 mL of water, and 30 mL of 6 M hydrochloric acid was added for 2 h reflux. Post-reflux, the mixture was cooled under tap water. The hydrolysate was neutralized with 40% NaOH solution and 6 M hydrochloric acid, using methyl red as the indicator. To precipitate proteins, pectin, and other contaminants, 20 mL of 20% neutral lead acetate solution was introduced, followed by the addition of 20 mL of 10% NaSO_4_ solution to eliminate excess lead. The total glucose content was assessed via the glucose oxidase–peroxidase (GOPOD) technique, and the total starch content was derived using the formula provided. The following two formulas were used to calculate the total starch content:

(3) $$TS\left(\%\right)=\frac{∆A\times F\times FV\times 0.9}{W}$$

(4) $$F=\frac{100(ug\;of\;D-glucose)}{absorbance\;for\;100\;ug\;of\;glucose}$$

where Δ*A* is the absorbance (reaction) read against the reagent blank, *FV* is the final volume of the test solution (mL), and *W* is the weight (mg) of the sample taken for analysis.

### 2.8. In Vitro Digestibility of CMCS/CS/SA Microcapsules

The method by Englyst et al. [34] was adapted to evaluate the in vitro digestibility of CMCS/CS/SA microcapsules. Initially, trypsin (3 g) was mixed with 20 mL of deionized water for 10 min. Afterward, 15 mL of this mixture was moved to a centrifuge tube, to which amyloglucosidase (1.1 mL) was added. Next, 200 mg of microcapsules and 18 mL of an acetate buffer (pH 5.20) were introduced into the tube, which was then placed in a boiling water bath for 30 min to gel. Upon cooling to 37 °C, 20 glass microcapsules and 2 mL of enzyme blend were added per tube, followed by incubation at 37 °C in a shaking water bath. Incubation times were set at 0, 20, 60, 90, 120, and 180 min, after which, a 0.1 mL sample of the hydrolysis product was taken from each tube, mixed with 0.9 mL of a 90% ethanol solution, and centrifuged. The glucose concentration in the supernatant was quantified using K-GLUC reagent. To mimic the human gastrointestinal environment, hydrolysis and digestion curves were modeled using first-order kinetics, allowing for the calculation of rapidly digestible starch (%RDS) and slowly digestible starch (%SDS) using predefined formulas.

%RDS = (G20 − G0) × 0.9 × 100/S(5)

%SDS = (G120 − G20) × 0.9 × 100/S(6)

%RS = [TS − (RDS + SDS)] × 100/S(7)

G0, G20, and G120 indicate the amounts of glucose in the hydrolysate at 0, 20, and 120 min, respectively (in mg), following the hydrolysis of the microcapsules with amyloglucosidase and pancreatin, while S refers to the overall starch content in the microcapsules (mg).

The human body’s gastrointestinal conditions were mimicked, and the starch digestion and hydrolysis progression conformed to a first-order kinetic formula:

C = 1 − e ^−kt^ (8)

C (%) represents the percentage of starch digested at time t (min), while 1 − C denotes the fraction of starch that remains undigested after time t. The coefficient k (min^−1^) indicates the rate of digestion. To determine k, a linear least square fitting of Equation (8)’s solution was performed. The linearity of this plot serves as an indicator of the suitability of applying first-order kinetics.

### 2.9. Statistical Analysis

All tests were performed in triplicate or more. The results are expressed as mean ± standard deviation. Variance analysis was carried out with ANOVA through IBM SPSS Statistics 23 (IBM, Armonk, NY, USA). Significant discrepancies were identified with Ducan’s multiple range tests at a 95% confidence interval (*p* < 0.05).

## 3. Results

### 3.1. Microcapsule Preparation Process for CMCS/CS/SA Microcapsules

**Figure 1:**
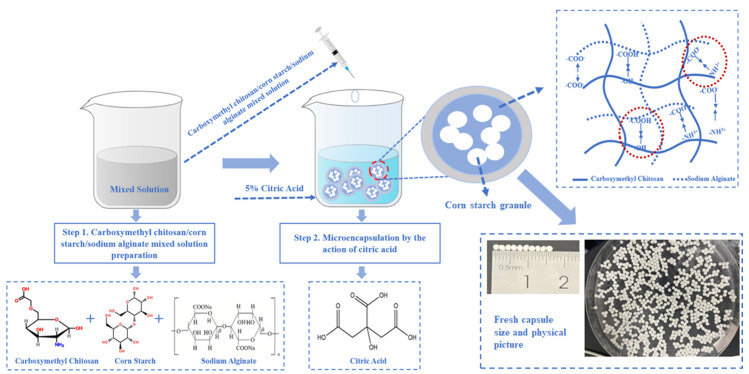
Microcapsule preparation process.

The fabrication process of CMCS/CS/SA microcapsules is depicted in Figure 1. These microcapsules were prepared by introducing the CMCS/CS/SA solution into a CA solution. This process resulted in the rapid precipitation of regular and smooth spheres from the solution, facilitated by hydrogen bonds and electrostatic interactions among CA, CMCS, and SA. When CMCS alone was added, the interaction force was weak, and a dense gel network did not form. However, upon the addition of SA to the CMCS system, the gel network became progressively more compact. This was attributed to the formation of strong interactions between CMCS, SA and CA, including hydrogen bonding between -COOH and -OH groups and electrostatic interactions between -NH^3+^ and -COO^−^ groups. The concentration of SA influenced the strength of these interactions, with higher SA concentrations leading to stronger interactions and a more compact gel network, consistent with the findings of Jing et al. [30].

### 3.2. Gel Texture Properties of CMCS/CS/SA Microcapsules

Table 1 illustrates the textural characteristics of both uncooked and cooked CMCS/CS/SA microcapsules. Uncooked CMCS/CS/SA_0%_ microcapsules demonstrated a hardness of 467.76 ± 14.69 g, attributed to the network structure formed by CMCS and CA through hydrogen bonding, consistent with findings by Zhou et al. [35,36], who demonstrated similar gel networks using carboxymethyl chitosan and tannic acid for wound dressing.

The incorporation of SA increased the hardness of uncooked CMCS/CS/SA microcapsules from 694.58 ± 15.62 g to 1519.02 ± 14.69 g as the concentration rose from 0.5% to 2%. The addition strengthened the gel network formed by CMCS and CA, primarily through enhanced hydrogen bonding and electrostatic interactions, leading to an improved density and hardness of the gel structure. Huang et al. observed similar effects, indicating that SA, CMCS, and gelatin could form a dense gel network through hydrogen bonding and electrostatic interaction, thereby enhancing mechanical properties [36].

Furthermore, cooking substantially increased the hardness of CMCS/CS/SA microcapsules. Boiled CMCS/CS/SA_0%_ microcapsules exhibited a hardness of 964.16 ± 31.24 g, significantly higher than the unboiled control. Following cooking, all microcapsules displayed increased hardness, peaking at 4230.30 ± 1.45 g with 2.5% SA concentration. This phenomenon likely resulted from starch granule gelatinization during cooking, followed by retrogradation to form a gel, thereby filling the bead’s network structure and increasing its hardness. Additionally, Cui et al. [28] observed increased hardness in cooked calcium alginate potato microcapsules due to starch gelatinization, consistent with our findings.

### 3.3. Microstructure of CMCS/CS/SA Microcapsules

**Figure 2:**
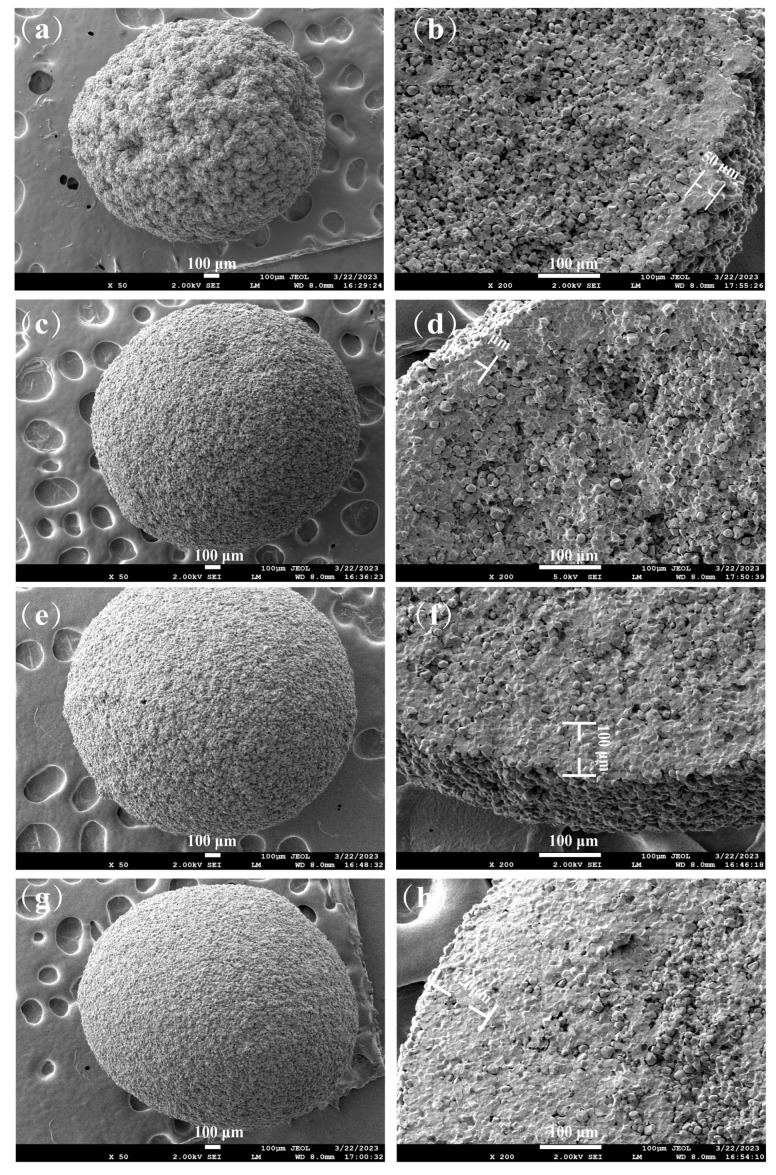
Surface and cross-section SEM images of CMCS/CS/SA microcapsules with different SA concentrations. (**a**,**c**,**e**,**g**) Surface images of microcapsules with sodium alginate concentrations of 0.5%, 1%, 1.5%, and 2%, respectively. (**b**,**d**,**f**,**h**) are cross-section images of microcapsules with sodium alginate concentrations of 0.5%, 1%, 1.5%, and 2%, respectively.

The SEM images in Figure 2 depict microcapsules with varying ratios of CMCS/CS/SA_0.5%_, CMCS/CS/SA_1%_, CMCS/CS/SA_1.5%_, and CMCS/CS/SA_2%_. Notably, the CMCS/CS/SA_0%_ sample lost its shape during lyophilization, precluding SEM analysis.

At a low SA concentration (0.5%), the CMCS/CS/SA_0.5%_ microcapsules exhibited a rougher surface with a more pronounced hole distribution. This roughness likely stemmed from the less compact gel network formed due to the low SA concentration, resulting in the incomplete encapsulation of starch within the beads. Conversely, as the concentration of SA increased from 1.5% to 2%, the surface of the beads gradually became smoother, devoid of obvious holes. This indicates that higher SA concentration enhanced the gel network’s structural integrity, resulting in denser networks that better enveloped the starch.

A cross-section analysis (Figure 2b,d,f,h) revealed notable differences in the shell thickness and internal network structure across microcapsules with varying SA concentrations. At 0.5% SA concentration, the shell exhibited non-uniform thickness, approximately 50 μm, with evident pores in the internal network structure, suggesting a looser gel network structure formed by CMCS and SA. With increasing SA concentration, the microcapsule shell’s edge became clearer, and the internal network structure formed by SA and CMCS became denser, with reduced pore formation. Shell thickness increased with rising SA concentration, measuring approximately 50 μm, 90 μm, 100 μm, and 120 μm for CMCS/CS/SA_0.5%_, CMCS/CS/SA_1%_, CMCS/CS/SA_1.5%_, and CMCS/CS/SA_2%_, respectively. This indicates that higher SA concentrations led to denser gel network structures, attributed to enhanced hydrogen bonding and electrostatic interactions within the CMCS/CS/SA gel network. Xie et al. [37] demonstrated a similar enhancement in gel strength by introducing SA into a gel matrix composed of oxalic acid and CMCS, highlighting the role of hydrogen bonding and electrostatic interactions.

### 3.4. Swelling Power and Solubility of CMCS/CS/SA Microcapsules

Table 2 illustrates the swelling and solubility characteristics of CS and four types of CMCS/CS/SA microcapsules at temperatures ranging from 55 °C to 95 °C for 30 min. Both the swelling power of CS and CMCS/CS/SA microcapsules increased with rising temperature. Specifically, the swelling power of CS escalated from 2.92 ± 0.5 g/g to 15.28 ± 0.21 g/g as the temperature increased from 55 °C to 95 °C. Similarly, the swelling of CMCS/CS/SA_2%_ microcapsules rose from 2.03 ± 0.13 g/g to 3.76 ± 0.66 g/g over the same temperature range. This trend suggests that water ingress into the microcapsules accelerated at higher temperatures.

Moreover, at a given temperature, the swelling degree of microcapsules decreased with increasing SA concentration. For instance, at 95 °C, the swelling degree of CMCS/CS/SA microcapsules dropped from 5.87 ± 0.57 g/g to 3.76 ± 0.66 g/g as the SA concentration increased from 0.5% to 2%. Compared to CMCS/CS/SA_0.5%_, CMCS/CS/SA_2%_ could form a denser network and thicker microcapsule shells due to stronger hydrogen bonds and electrostatic interactions, as confirmed by the SEM results showing increased shell thickness. This mesh-like network structure effectively prevented water from entering the microcapsules, thereby inhibiting the swelling behavior of starch within. The higher the SA concentration, the denser the gel network formed, preventing internal starch from contacting water and thus exerting a stronger inhibitory effect on microcapsule expansion. Feltre et al. [38] observed that CS embedded with calcium alginate was less absorbent than natural CS, attributed to the protective effect of the calcium alginate microsphere shell.

Regarding solubility, as the temperature increased, the solubility of the samples gradually rose. For instance, when the temperature ranged from 55 °C to 95 °C, the solubility of CS increased from 0.6 ± 0.1 to 7.6 ± 0.12, and for CMCS/CS/SA_2%_, it increased from 6.75 ± 0.65 to 9.05 ± 0.11. However, at the same temperature, the solubility of microcapsules decreased as the SA concentration increased. For example, at 95 °C, as the SA concentration rose from 0.5% to 2%, the solubility decreased from 13.15 ± 0.13 to 9.05 ± 0.11. This reduction in the solubility of CS granules from the microcapsules can be attributed to a denser gel network structure, which was reinforced by stronger hydrogen bonds and electrostatic interactions at higher SA concentrations. Qin et al. [33] demonstrated that using calcium chloride to encapsulate degraded starch in SA microspheres resulted in the reduced solubility of the starch microspheres, suggesting that high SA concentrations could generate strong static electricity and form a dense gel network to decrease solubility.

### 3.5. DSC of CMCS/CS/SA Microcapsules

The onset temperature (*T*_o_), peak temperature (*T*_p_), conclusion temperature (*T*_c_), and enthalpy change (*ΔH*) of CMCS/CS/SA microcapsules are presented in Table 3. For CS, *T*_o_, *T*_p_, *T*_c_, and *ΔH* were recorded as 68.03 ± 0.73 °C, 71.91 ± 0.51 °C, 71.91 ± 0.51 °C, and −14.06 ± 0.08 J·g^−1^, respectively. As the SA concentration increased from 0.5% to 2%, *T*_o_ rose by approximately 3.62 °C, *T*_p_ by about 4.41 °C, *T*_c_ by roughly 7.81 °C, and *ΔH* decreased by around 3.82 J·g^−1^. The higher SA concentrations resulted in a denser gel network, requiring more thermal energy for starch gelatinization within the beads. With SA added, intermolecular hydrogen bonds and electrostatic interactions were reinforced, forming a compact network structure that hindered water flow in the microcapsules, thereby improving the thermal properties of the internal starch granules. The *ΔH* in DSC is an exothermic reaction by starch during gelatinization. Cooke et al. [39] demonstrated that the starch’s *ΔH* indicated the extent of starch granule loss and expansion. The microcapsule shell acted as a physical barrier, reducing water absorption during starch gelatinization, resulting in incomplete starch gelatinization, reduced heat absorption, and a consequently lower *ΔH* of gelatinization. Yang et al. [40] noted that the shell of calcium carbonate starch gel beads protected internal starch granules, leading to significantly higher *T*_o_, *T*_p_, and *T*_c_ compared to natural pea starch, as shown in the DSC results.

### 3.6. Starch Digestion Curves for CMCS/CS/SA Microcapsules

**Figure 3:**
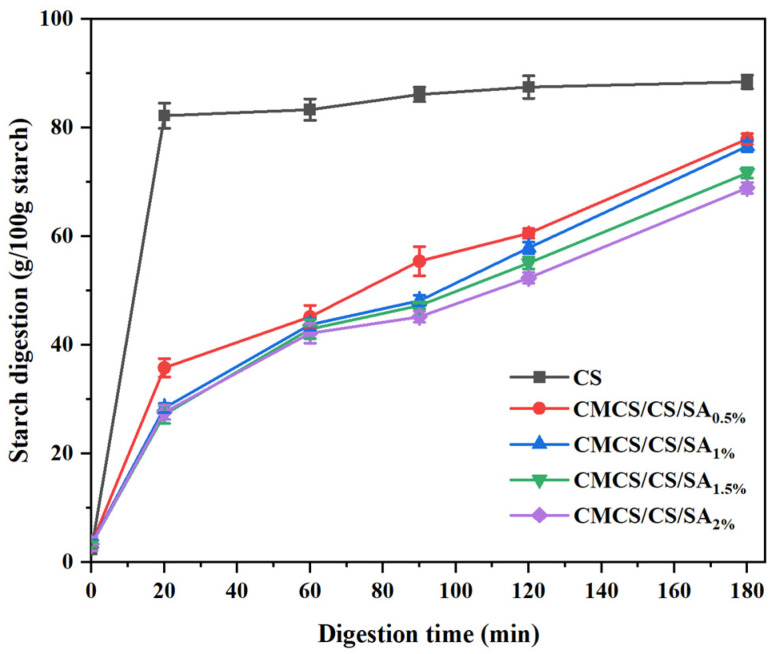
In vitro digestibility of CMCS/CS/SA microcapsules with different SA concentrations.

The in vitro digestive kinetics of CS and sample microcapsules at different time intervals are illustrated in Figure 3. Compared with corn starch, the construction of the shell significantly reduced the digestion rate of starch, and the difference between CMCS/CS/SA_0.5%_ and CMCS/CS/SA_2%_ was significant. CS exhibited a starch digestibility of 82.254% at 20 min and 87.690% at 120 min, with the rate of starch digestion remaining relatively constant thereafter. In comparison, the starch hydrolysis rate of CMCS/CS/SA microcapsule samples decreased. With the SA concentration increasing from 0.5% to 2%, the digestibility of CMCS/CS/SA microcapsules decreased from 35.818% to 27.649% at 20 min and from 77.969% to 68.961% at 180 min. It was noted that as the SA concentration varied in CMCS/CS/SA microcapsules, an increase in SA concentration correlated with a decrease in the digestion rate of the microcapsules. At low SA concentrations, the network structure formed was weak, making it challenging to effectively resist amylase hydrolysis. Conversely, with higher SA concentrations, the network between SA and CMCS strengthened, forming a more effective barrier against amylase, thus impeding further starch hydrolysis and reducing the digestion rate. Park et al. [41] demonstrated that microspheres formed by CA and calcium chloride could elevate the RS content of waxy CS in starch microspheres.

### 3.7. In Vitro Digestion of CMCS/CS/SA Microcapsules

Table 4 displays the contents of the RDS, SDS, and RS of CS and the four prepared microcapsules during in vitro digestion. After a 30 min boiling water bath, the contents of the RDS, SDS, and RS of CS were 83.86% ± 0.76%, 7.59% ± 0.06%, and 8.54% ± 0.14%, respectively. In addition, with the concentration of SA in CMCS/CS/SA microcapsules increasing from 0.5% to 2%, the RDS content notably decreased from 25.86% ± 0.13% to 20.06% ± 0.56% (*p* < 0.05); SDS content significantly decreased from 31.76% ± 0.22% to 22.28% ± 0.21% (*p* < 0.05); RS content increased from 42.37% ± 0.07% to 57.65% ± 0.45% (*p* < 0.05). The addition of SA during microcapsule preparation significantly reduced RDS (*p* < 0.05), while SDS and RS contents were notably increased (*p* < 0.05). These findings suggest that the gel network formed by crosslinking CMCS and SA under the influence of the CA crosslinking agent inhibited starch hydrolysis by amylase. Similarly, Cui et al. [28] reported that SA, acting as an encapsulation film of starch granules, could prevent amylase from hydrolyzing starch and decrease the RS content of CS in microspheres to 29.39%. When the SA concentration reached 2%, the hydrogen bond and electrostatic interaction of the gel network formed by CMCS, SA, and CA were at their strongest, resulting in the densest network structure, which better shielded the contact between starch and the enzyme. Therefore, starch microcapsules composed of CMCS/CS/SA represent a practical approach to enhancing starch digestion resistance. Qin et al. [33] observed that the calcium alginate shell prevented amylase from contacting and hydrolyzing starch during in vitro digestion, leading to an increase in RS in starch microspheres with increasing SA concentration.

### 3.8. SEM during In Vitro Digestion

**Figure 4:**
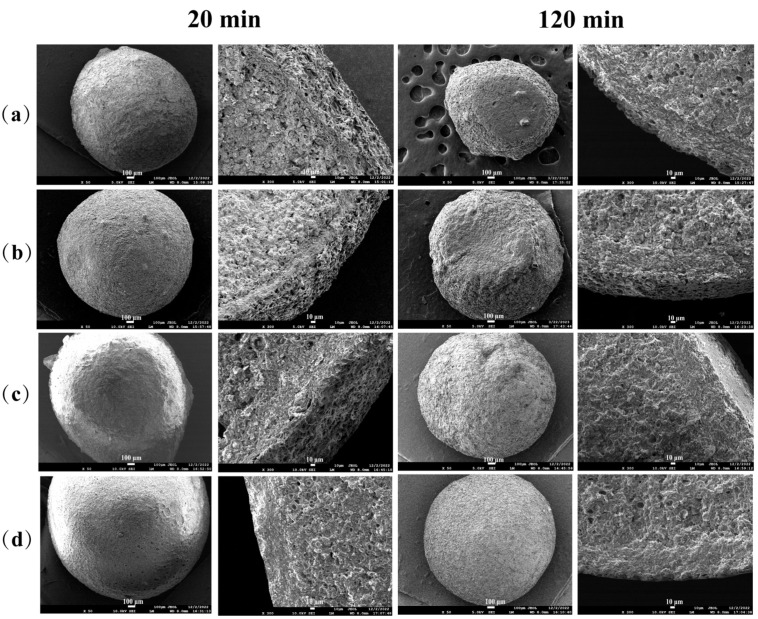
SEM images of surface and cross section of four SA concentration CMCS/CS/SA microcapsules after digestion for 20 min and 120 min. (**a**–**d**) are surface and cross-section images of microcapsules with SA concentrations of 0.5%, 1%, 1.5%, and 2%, respectively.

The surface and cross-section SEM images of the four prepared microcapsules after 20 min and 120 min of in vitro digestion are presented in Figure 4. As the hydrolysis time increased from 20 min to 120 min, the microcapsules visibly decreased in volume, and the degree of surface shrinkage decreased, likely due to the expulsion of internal starch from pores. During the digestion process, amylase could penetrate the microcapsules through pores generated during in vitro digestion, hydrolyze starch, and subsequently, upon lyophilization, the volume of microcapsules decreased. With the increase in SA concentration, the network structure of the microcapsules became denser, and higher concentrations of SA resulted in thicker microcapsule walls, effectively preventing the entry of starch-digesting enzymes and the dissolution of starch, thereby helping to maintain the original spherical appearance. Additionally, the cross-sectional diagrams reveal that as digestion progressed, the internal pores of the 120 min microcapsules were more pronounced than those of 20 min microcapsules, likely due to the porous structure left by amylase hydrolyzing starch. Despite digestion, the walls of the microcapsules formed by CMCS/CS/SA remained clearly visible and intact at the edges, indicating that the wall material formed by CMCS/CS/SA could withstand digestion, demonstrating a good anti-digestion effect and potential use as an anti-digestion wall material. Microcapsules with higher concentrations of SA also exhibited denser and thicker shells. The slower the structural changes observed on the surface and interior, the slower the hydrolysis rate of starch, corresponding to the digestion curve, highlighting the crucial role of the shell in resisting amylase hydrolysis. Li et al. [42] confirmed that short-chain amylose coated by SA microspheres was effectively protected during gastrointestinal digestion, with the calcium alginate shell playing a crucial role in preventing digestive enzymes from entering the microspheres.

## 4. Conclusions

In our study, we prepared microcapsules using SA, CMCS, and CA as raw materials, employing physical crosslinking methods like hydrogen bonding by -COOH and -OH groups and electrostatic interaction by -NH^3+^ and -COO^−^ groups. The transparent shell formed by CMCS/CS/SA effectively enveloped CS within the microcapsule, reducing the exposure to water molecules and digestive enzymes, and thereby protecting the starch granules. At 95 °C, the swelling degree of CMCS/CS/SA_2%_ was 2.03 ± 0.13 g/g, which was 11.52 g/g lower than that of CS. The *T*_o_, *T*_p_, and *T*_c_ of CMCS/CS/SA microcapsules were increased compared to CS, and the *ΔH* of CMCS/CS/SA_2%_ was −5.96 ± 0.12 J·g^−1^, indicating the excellent thermal stability of the microspheres. With an increase in SA concentration from 0.5% to 2%, the RS content of CMCS/CS/SA microcapsules increased from 42.37 ± 0.07% to 57.65 ± 0.45%, respectively. These microcapsules demonstrated the ability to delay starch digestion and maintain the stability of postprandial blood sugar levels; it is of great significance to alleviate and prevent chronic diseases related to blood glucose metabolism problems.

## Figures and Tables

**Table 1:** Structural characteristics of fresh CMCS/CS/SA microcapsules and boiled microcapsules.

	Hardness (g)	Adhesiveness (g·s)	Springiness	Cohesiveness	Gumminess	Chewiness
Sample						
CMCS/CS/SA_0%_	467.76 ± 14.69 ^e^	−0.65 ± 0.02 ^a^	1.23 ± 0.06 ^a^	1.02 ± 0.02 ^a^	1147.54 ± 13.65 ^a^	1002.67 ± 8.36 ^a^
CMCS/CS/SA_0.5%_	694.58 ± 15.62 ^d^	−4.32 ± 0.14 ^e^	0.60 ± 0.02 ^e^	0.60 ± 0.02 ^e^	421.17 ± 23.06 ^e^	250.50 ± 5.36 ^e^
CMCS/CS/SA_1%_	1119.31 ± 21.21 ^c^	−2.26 ± 0.21 ^d^	0.64 ± 0.04 ^d^	0.63 ± 0.01 ^d^	698.29 ± 18.71 ^d^	452.04 ± 35.77 ^d^
CMCS/CS/SA_1.5%_	1308.64 ± 10.16 ^b^	−0.80 ± 0.00 ^b^	0.81 ± 0.00 ^c^	0.73 ± 0.00 ^b^	880.66 ± 0.79 ^c^	756.52 ± 0.49 ^c^
CMCS/CS/SA_2%_	1519.02 ± 14.69 ^a^	−1.24 ± 0.05 ^c^	0.90 ± 0.00 ^b^	0.71 ± 0.01 ^c^	1075.56 ± 15.12 ^b^	969.81 ± 6.17 ^b^
Sample (after cooking)						
CMCS/CS/SA_0%_	964.16 ± 31.24 ^e^	−0.55 ± 0.02 ^a^	1.31 ± 0.02 ^a^	1.06 ± 0.01 ^a^	1727.94 ± 16.15 ^a^	1711.71 ± 18.21 ^a^
CMCS/CS/SA_0.5%_	1865.67 ± 37.56 ^d^	−3.19 ± 0.36 ^c^	0.71 ± 0.00 ^d^	0.64 ± 0.01 ^c^	1195.92 ± 40.36 ^d^	859.34 ± 22.08 ^d^
CMCS/CS/SA_1%_	3601.87 ± 12.11 ^c^	−1.23 ± 0.02 ^b^	0.76 ± 0.01 ^b^	0.65 ± 0.01 ^c^	2389.27 ± 3.01 ^c^	1824.82 ± 5.47 ^b^
CMCS/CS/SA_1.5%_	3858.92 ± 4.33 ^b^	−0.57 ± 0.01 ^a^	0.73 ± 0.00 ^c^	0.76 ± 0.01 ^a^	2982.78 ± 5.90 ^b^	1732.87 ± 5.96 ^c^
CMCS/CS/SA_2%_	4230.30 ± 1.45 ^a^	−0.44 ± 0.04 ^a^	0.88 ± 0.06 ^a^	0.72 ± 0.01 ^b^	3070.59 ± 2.02 ^a^	2718.81 ± 3.15 ^a^

Values represent the means ± standard deviations of triplicate tests. Values with different letters (a, b, c, d and e) are significantly different (*p* < 0.05). CMCS: carboxymethyl chitin; CS: corn starch; SA: sodium alginate; 2%, 1.5%, 1%, and 0.5% represent the concentration of sodium alginate, respectively.

**Table 2:** Solubility and swelling power of CMCS/CS/SA microcapsules.

Temperature(°C)	CS	CMCS/CS/SA_0.5%_	CMCS/CS/SA_1%_	CMCS/CS/SA_1.5%_	CMCS/CS/SA_2%_
Swelling power (g/g)					
95 °C	15.28 ± 0.21 ^a^	5.87 ± 0.57 ^b^	4.19 ± 0.18 ^c^	4.24 ± 0.64 ^c^	3.76 ± 0.66 ^c^
85 °C	11.36 ± 0.32 ^a^	5.19 ± 0.15 ^b^	3.89 ± 0.65 ^c^	4.15 ± 0.12 ^cd^	3.36 ± 0.32 ^d^
75 °C	10.57 ± 0.37 ^a^	4.62 ± 0.42 ^b^	3.82 ± 0.31 ^c^	3.40 ± 0.20 ^cd^	3.13 ± 0.12 ^d^
65 °C	3.22 ± 0.21 ^a^	2.92 ± 0.42 ^a^	2.89 ± 0.81 ^a^	2.42 ± 0.22 ^a^	2.30 ± 0.10 ^a^
55 °C	2.92 ± 0.50 ^a^	2.32 ± 0.31 ^ab^	2.29 ± 0.25 ^b^	2.11 ± 0.11 ^b^	2.03 ± 0.13 ^b^
Solubility (%)					
95 °C	7.6 ± 0.12 ^e^	13.15 ± 0.13 ^a^	12.05 ± 0.45 ^b^	10.35 ± 0.25 ^c^	9.05 ± 0.11 ^d^
85 °C	3.43 ± 0.21 ^c^	10.98 ± 0.66 ^a^	9.96 ± 0.53 ^a^	5.79 ± 0.71 ^b^	5.87 ± 0.53 ^b^
75 °C	3.60 ± 0.20 ^e^	10.05 ± 0.25 ^a^	8.15 ± 0.52 ^b^	4.65 ± 0.85 ^d^	6.00 ± 0.20 ^c^
65 °C	0.98 ± 0.08 ^e^	8.65 ± 0.24 ^a^	6.05 ± 0.32 ^b^	5.60 ± 0.30 ^d^	5.85 ± 0.25 ^c^
55 °C	0.60 ± 0.10 ^d^	8.15 ± 0.35 ^a^	7.45 ± 0.41 ^ab^	5.95 ± 0.66 ^c^	6.75 ± 0.65 ^bc^

Values represent the means ± standard deviations of triplicate tests. Values with different letters (a, b, c, d and e) are significantly different (*p* < 0.05). CMCS: carboxymethyl chitin; CS: corn starch; SA: sodium alginate; 2%, 1.5%, 1%, and 0.5% represent the concentration of sodium alginate, respectively.

**Table 3:** The onset, peak, and conclusion temperatures (*T*_o_, *T*_p_, and *T*_c_) and enthalpy change (*ΔH*) of native starch and microcapsules.

Sample	*T*_o_/°C	*T*_p_/°C	*T*_c_/°C	*ΔH*/J·g^−1^
CS	68.03 ± 0.73 ^c^	71.91 ± 0.51 ^d^	76.13 ± 0.33 ^e^	−14.06 ± 0.08 ^e^
CMCS/CS/SA_0.5%_	69.27 ± 0.25 ^b^	73.95 ± 0.74 ^c^	79.65 ± 0.53 ^d^	−9.78 ± 0.21 ^d^
CMCS/CS/SA_1%_	70.33 ± 0.31 ^b^	74.86 ± 0.66 ^c^	81.31 ± 0.21 ^c^	−7.54 ± 0.17 ^c^
CMCS/CS/SA_1.5%_	71.78 ± 0.66 ^a^	76.03 ± 0.53 ^b^	84.75 ± 0.54 ^b^	−6.33 ± 0.21 ^b^
CMCS/CS/SA_2%_	72.89 ± 0.81 ^a^	78.36 ± 0.26 ^a^	86.17 ± 0.34 ^a^	−5.96 ± 0.12 ^a^

Values represent the means ± standard deviations of triplicate tests. Values with different letters (a, b, c, d and e) are significantly different (*p <* 0.05). CMCS: carboxymethyl chitin; CS: corn starch; SA: sodium alginate; 2%, 1.5%, 1%, and 0.5% represent the concentration of sodium alginate, respectively.

**Table 4:** The RDS, SDS and RS contents of native starch and microcapsules.

Sample	RDS (%)	SDS (%)	RS (%)
CS	83.86 ± 0.76 ^a^	7.59 ± 0.06 ^e^	8.54 ± 0.14 ^e^
CMCS/CS/SA_0.5%_	25.86 ± 0.13 ^b^	31.76 ± 0.22 ^a^	42.37 ± 0.07 ^d^
CMCS/CS/SA_1%_	24.43 ± 0.21 ^c^	26.92 ± 0.32 ^b^	48.62 ± 0.42 ^c^
CMCS/CS/SA_1.5%_	21.83 ± 0.33 ^d^	25.13 ± 0.33 ^c^	53.03 ± 0.51 ^b^
CMCS/CS/SA_2%_	20.06 ± 0.56 ^e^	22.28 ± 0.21 ^d^	57.65 ± 0.45 ^a^

Values represent the means ± standard deviations of triplicate tests. Values with different letters (a, b, c, d and e) are significantly different (*p* < 0.05). CMCS: carboxymethyl chitin; CS: corn starch; SA: sodium alginate; 2%, 1.5%, 1%, and 0.5% represent the concentration of sodium alginate, respectively.

## Data Availability

The original contributions presented in the study are included in the article; further inquiries can be directed to the corresponding author.
